# The Unusual Suspects in Cytokinesis: Fitting the Pieces Together

**DOI:** 10.3389/fcell.2020.00441

**Published:** 2020-06-18

**Authors:** Ly T. S. Nguyen, Douglas N. Robinson

**Affiliations:** ^1^Department of Cell Biology, Johns Hopkins School of Medicine, Baltimore, MD, United States; ^2^Department of Pharmacology and Molecular Sciences, Johns Hopkins School of Medicine, Baltimore, MD, United States; ^3^Department of Medicine, Johns Hopkins School of Medicine, Baltimore, MD, United States; ^4^Department of Oncology, Johns Hopkins School of Medicine, Baltimore, MD, United States; ^5^Chemical and Biomolecular Engineering, Johns Hopkins University Whiting School of Engineering, Baltimore, MD, United States

**Keywords:** discoidin, chloride intracellular channels, importins, Ran, helicases, RNP, mmsdh

## Abstract

Cytokinesis is the step of the cell cycle in which the cell must faithfully separate the chromosomes and cytoplasm, yielding two daughter cells. The assembly and contraction of the contractile network is spatially and temporally coupled with the formation of the mitotic spindle to ensure the successful completion of cytokinesis. While decades of studies have elucidated the components of this machinery, the so-called *usual suspects*, and their functions, many lines of evidence are pointing to other unexpected proteins and sub-cellular systems as also being involved in cytokinesis. These we term the *unusual suspects*. In this review, we introduce recent discoveries on some of these new unusual suspects and begin to consider how these subcellular systems snap together to help complete the puzzle of cytokinesis.

## Overview of Cytokinesis

The growth and development of cells and tissues depend in part on the segregation of genetic and cytoplasmic components of a mother cell into two daughter cells during cell division. Defects in any step of this process can result in missegregation of genetic material, leading to multinucleation, polyploidy, and eventually aneuploidy, which is associated with many diseases, including cancer (Storchova and Pellman, 2004; Normand and King, 2010). The final step of cell cycle is cytokinesis, when a contractile network (CN) composed of actin filaments, non-muscle myosin II and other necessary factors work to physically divide the mother cell (Fujiwara et al., 2005; Shi and King, 2005; Robinson et al., 2012; Leite et al., 2019; Pollard and O’Shaughnessy, 2019). The CN is the driver and responder to active force production and can be organized into meshworks like those in mammalian and amoeboid cells or rings like those in yeasts (Fishkind and Wang, 1993; Kamasaki et al., 2007; Reichl et al., 2008). Furthermore, the fluid dynamics of the system that are created in part by cortical tension and the cell’s viscoelastic properties are also major contributors to cytokinesis (Zhang and Robinson, 2005). Collectively, furrow ingression is driven by active (ATP consuming) contraction from myosin II pulling on actin filaments and the Laplace pressures created by cortical tension and local (cleavage furrow) and global/polar (emerging daughter cell) membrane curvatures (Zhang and Robinson, 2005; Poirier et al., 2012).

The process of the CN formation is spatially and temporally coupled with the assembly of the antiparallel, interdigitating microtubules, which can be formed either by the astral microtubules or the central spindle (Baker et al., 1993; Ding et al., 1993). The central spindle plays multiple roles during cell division, including segregation of chromosomes, positioning of the cleavage furrow, and separation of daughter cells (Straight and Field, 2000). Cytokinesis is completed by the process of abscission, facilitated by the ESCRT-III complexes, resulting in complete physical separation of the two daughter cells (Ettema and Bernander, 2009; Mierzwa and Gerlich, 2014).

**FIGURE 1:**
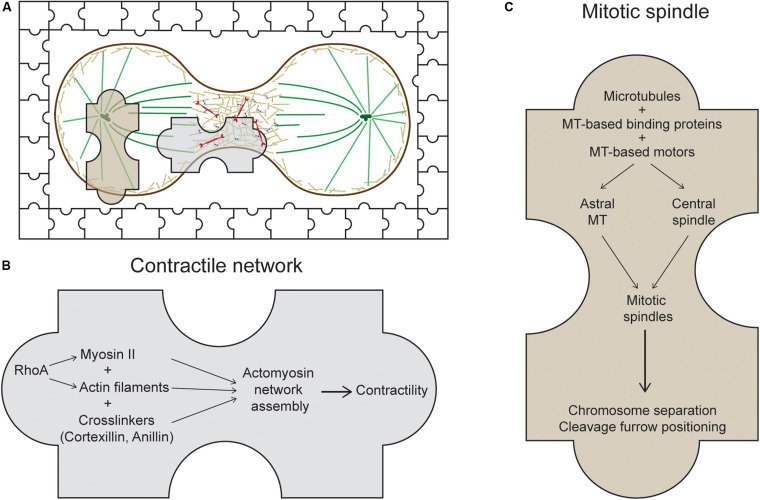
The *usual suspects* of cytokinesis. **(A)** During cytokinesis, the cell cortex contracts at the midzone forming the cleavage furrow. The system at work is presented as a jigsaw puzzle with multiple pieces coming together. Many players of this process can be categorized into two main sets of machinery: the contractile network (CN) and the mitotic spindle, represented by the two large jigsaw pieces. **(B)** The CN consists of actin filaments, myosin II, and actin crosslinkers, which are organized into meshworks or rings and which generate mechanical forces at the cleavage furrow. One major regulator of this CN in metazoans is the RhoA small GTPase (Chircop, 2014). **(C)** The mitotic spindle is composed of antiparallel, interdigitating microtubules that form either the astral microtubules or the central spindle. Along with its binding proteins and motors, the mitotic spindle segregates the chromosomes, positions the cleavage furrow, and helps regulate daughter cell separation.

While it has been appreciated for decades that cytokinesis is a highly robust and dynamic process, we are beginning to understand some of the mechanisms responsible for this robustness (Kee et al., 2012; Srivastava and Robinson, 2015; Srivastava et al., 2016; Singh et al., 2019). Crosstalk between and integration of various sub-cellular systems exist to ensure fidelity and coordination of the entire cellular system (Kothari et al., 2019a). The field has appropriately focused on what we will term the *usual suspects* – i.e., the players of actomyosin II CN, microtubule-based mitotic spindles, RhoGTPase regulators, and many others (Figure 1). However, many lines of evidence are pointing to many unexpected proteins and sub-cellular systems as also being involved in cytokinesis – we term these proteins the *unusual suspects*.

In this review, we focus on recent discoveries implicating some of these new unusual suspects- factors and proteins involved in pathways and networks that are generally assigned different sub-cellular roles, but that appear to be contributing to the cytokinesis machinery too. These unusual suspects are categorized into a total of four groups: membrane-associated proteins, RNA-related proteins, nuclear proteins, and metabolic enzymes. We anticipate that embracing these new players will lead the field to uncover a more holistic understanding of how cytokinesis works. But, perhaps more importantly, they will also guide us to a deeper understanding of how cellular systems are integrated in complex biological processes more broadly. Moreover, we want to state up front that we believe that this list of unusual suspects is just beginning to take off, and therefore, we expect that the list presented here is neither comprehensive nor final.

## Membrane-Associated Proteins

### Discoidin

Discoidin I and II are *N*-acetylgalactosamine (GalNAc)-binding lectins found predominantly in the cytoplasm of the amoeba *Dictyostelium discoideum* (Alexander et al., 1992). Classically, the discoidins are considered key to *Dictyostelium* development as their expression increases as the *Dictyostelium* cells enter the developmental stage (Rosen et al., 1973; Frazier et al., 1975). Originally, discoidins were suggested to play a role in mediating cell–cell interaction owing to their lectin properties. However, Discoidin I was recently found to interact genetically and biochemically with a key protein involved in cell contractility and cytokinesis, namely cortexillin I (Robinson and Spudich, 2000; Kothari et al., 2019b). Myosin II and the actin crosslinker Cortexillin I are essential components of the CN and their mechanosensory ability and accumulation at the cleavage furrow were proven to be vital for the progression and completion of cytokinesis (Faix et al., 2001; Zhang and Robinson, 2005; Effler et al., 2007; Kee and Robinson, 2008; Ren et al., 2009). *In vivo* interactions of discoidin I and cortexillin I as well as IQGAP2, an activator of Cortexillin I and Myosin II, were also confirmed using fluorescence cross-correlation spectroscopy. Interestingly, discoidin also acts as a genetic suppressor of *cortexillin I* null mutants through a selection for genetic suppressors where discoidin overexpression could rescue the developmental phenotypes of *cortexillin I* null cells (Robinson and Spudich, 2000). Collectively, discoidin proteins might provide a scaffolding function and help recruit essential components of the CN system to the division site during cytokinesis.

Another possibility is that the discoidins may help to directly connect the CN to the plasma membrane. It is not fully understood in any system how the CN is attached to the membrane, particularly in the context of cytokinesis. Dividing *Dictyostelium* cells show an enrichment of glycosylated proteins at the cleavage furrow membrane as the lectin concanavalin A preferentially labels the cleavage furrow membrane (Faix et al., 2001). Thus, the combination of observations suggests a provocative hypothesis that extracellular discoidin might also interact with the cortical cytoskeleton through a transmembrane domain containing protein. The human discoidin domain receptor (DDR1) protein does exactly this, except it contains a transmembrane domain and interacts with non-muscle myosin IIA (Huang et al., 2009). Invasive tumors and associated metastases show elevated expression of DDR1, suggesting its role as a promoter of tumor cell invasion (Yang et al., 2010; Henriet et al., 2018). However, this type of function would most likely be distinct from discoidin’s function in forming complexes with cortexillin and IQGAP2 in the cytoplasm.

Discoidin was not the first sugar-related protein that was speculated to be crucial in cytokinesis-related processes. Mucin is also a glycosylated protein and is a component of ring canals and intercellular bridges, structures formed by incomplete cytokinesis, in various cell types and tissues in *Drosophila melanogaster* (Robinson and Cooley, 1997; Kramerova and Kramerov, 1999). Even though the exact functions of mucinoprotein in ring canal networks are not well-understood, it is possible that it might be an important constituent of a complex or backbone that facilitates the assembly of these structures. Further studies are needed to elaborate on the exact roles and functions of discoidin and other sugar-binding proteins that take part in cytokinesis.

### Chloride Intracellular Channels

Another unexpected class of membrane proteins that were found to play a role in cytokinesis are the highly-conserved chloride intracellular channels (CLICs). Comprised of six members that exist primarily as membrane-bound proteins, CLICs are involved in diverse cellular functions, including cell-cell interactions, angiogenesis, and membrane potential regulation (Rual et al., 2005; Singh and Ashley, 2007; Chalothorn et al., 2009). Multiple studies have also implicated CLIC4 in cancer progression. The expression level of CLIC4 was found to be gradually decreased in squamous cancer cells as they transformed from benign to malignant, a prominent indicator of a role in mitosis regulation (Suh et al., 2012).

Growing evidence implicates the function of CLIC4 in actin-mediated processes (Suginta et al., 2001; Singh et al., 2007; Singh, 2010). For instance, CLIC4 is recruited to the plasma membrane and the cleavage furrow upon activation of RhoA in an F-actin-dependent manner (Ponsioen et al., 2009; Argenzio et al., 2018). Additionally, CLIC4 is a direct molecular interactor of profilin-1 and a component in the RhoA-mDia2 signaling pathway that induces cortical actin polymerization (Argenzio et al., 2018). A previous proteomics study investigating the biochemical changes at the cell surface during cell division revealed a significant enrichment of both CLIC4 and CLIC1 on the surface of rounded up mitotic cells compared with flat interphase cells (Ozlu et al., 2015). A recent study comparing the interaction networks of CLIC4 and a CLIC4 mutant version defective in putative substrate binding led to the identification of ezrin, anillin, and ALIX, proteins known to be crucial for cytokinesis, as partners of CLIC4 (Uretmen Kagiali et al., 2020). Furthermore, double knockout of CLIC4 and CLIC1 subsequently impaired the stability of membrane-cortical actin interaction. HeLa cells lacking both CLIC4 and CLIC1 displayed abnormal bleb formation and defective abscission (Uretmen Kagiali et al., 2020). In another study, cells absent of CLIC4 had significantly decreased recruitment of myosin IIA and IIB to the furrow, similarly leading to membrane blebbing and impeding CN maturation (Peterman et al., 2020). It was also previously found that cortical actin controls cell cycle progression, and tumor cells lose their dependence on cortical branched actin (Molinie et al., 2019). Through their role in maintaining cortical and CN stability, CLICs might facilitate normal mitotic progression and prevent the transformation from benignity to malignancy in cancer cells.

Overall, CLIC4 and CLIC1 are two additional membrane-associated proteins that are recruited to the cortex, where they potentially help anchor the CN, allowing for proper contraction and cell division.

## RNA-Related Proteins

### RNA-Binding Proteins

RNPs (ribonucleoproteins) are a large family of RNA-binding proteins that have important roles in mRNA translation and regulation (Ziemienowicz et al., 2003). RNPs commonly localize in nuclei and cytoplasmic mRNP granules. In general, they contain one or more RNA-recognition motifs (RRMs) and often have a predicted intrinsically disorganized region (IDR). While the exact functions of many RNPs are still being defined, studies in some systems indicate that RNPs contribute to cytokinesis.

In *Dictyostelium*, overexpression of RNP-1A suppresses the effect of nocodazole, a microtubule inhibitor, on cellular growth of *Dictyostelium discoideum* (Zhou et al., 2010; Ngo et al., 2016). This protein has protective functions on the microtubule ends, and also localizes to the polar cortex in dividing cells and to the leading edges of migrating cells. These strongly suggest a role of RNP-1A in regulating microtubule dynamics, possibly through stabilizing the linkage between microtubules and the cortex. A later study using proteomics found that a separate RNP, RNP-1B, was a molecular interactor of cortexillin I (Kothari et al., 2019b). In the same study, using fluorescence cross-correlation spectroscopy, RNP-1A was found to interact strongly with cortexillin I. The full significance of these interactions is still under investigation.

The recognition of RNA-binding proteins as potential effectors of cytokinesis was not entirely surprising, as CAR-1, another RNA-binding protein found at P granules and the mitotic spindle during cell division, was identified using an RNA-interference screen for genes essential for cytokinesis in *C. elegans* embryos (Zipperlen et al., 2001; Boag et al., 2005; Squirrell et al., 2006). In *car-1*-depleted embryos, cleavage furrow ingression was significantly disrupted, and the anaphase spindle structure was greatly defective (Audhya et al., 2005). As the study was conducted primarily in early *C. elegans* embryos where tight temporal-spatial regulations of maternally supplied RNAs are critical, the defects found in *car-1*-depleted cells may be due to the local translational incompetence of microtubule components. This effect was similar to a previously characterized RNA-binding protein, CPEB, whose inhibition also results in spindle structural defects (Groisman et al., 2000). Overall, these roles of RNA-binding proteins in cytokinesis regulation elucidates an important crosstalk between local protein translation and steps in cell division. It is tempting to speculate that the unique localization and interactions of these RNP-1 proteins facilitates localized mRNA translation during cytokinesis when cells are undergoing dynamic and robust cell shape changes.

### RNA Helicases

Another unusual suspect that falls under the realm of RNA-related proteins are the RNA helicases. The importance of RNA helicases in regulating cell cycle progression and mitosis is not entirely surprising. Indeed, since the progression of the cell cycle requires the coordination of enormous cohorts of differentially expressed proteins through each step, cells need mechanisms to tightly regulate mRNA transcription, translation, and protein degradation to facilitate this dynamic process. For example, RNA helicases have been implicated to play critical roles in mRNA export pathways that are tightly linked to gene products involved in mitosis regulation (Strasser and Hurt, 2001; Herold et al., 2003). Indeed, the RNA helicases UAP56 and URH49 were characterized to form distinct mRNA export machineries to regulate a subset of genes specifically involved in mitosis (Yamazaki et al., 2010). Depletion of these RNA helicases, as a result, leads to mitotic progression defects.

However, unexpected functions of several RNA helicases in cytokinesis independent from its traditional roles in regulating mitotically expressed proteins have been discovered. CGH-1, a DEAD box RNA helicase that localizes to P granules and other possible mRNA–protein particles, was originally identified to be in a multiprotein complex together with CAR-1, another unusual suspect mentioned above, and a localization regulator of CAR-1 in *C. elegans* (Navarro et al., 2001; Audhya et al., 2005). Embryos depleted of *cgh-1* had penetrant sterility while those partially depleted exhibited perturbed localization of CAR-1 and phenocopied *car-1*-depleted cells with defects in microtubule structures. The study suggested that CGH-1 and CAR-1 cooperatively regulate anaphase spindle structure in embryonic cytokinesis.

Interestingly, CGH-1 is the *C. elegans* homolog of DDX6, a member of DEAD box RNA helicase proteins DDXs, which is involved diverse pathways, including mRNP assembly and export, immune response, regulation of cell cycle progression and tumorigenesis (Fuller-Pace, 2013). The exact functions of DDXs in regulating cell cycle and tumorigenesis remain controversial. Knockdown of DDX3 in mice embryonic cells led to reduced growth and proliferation (Li et al., 2014). However, a recent study elucidated a possible mechanism through which DDX3, another member of the DDX family, positively controls cytokinesis and subsequently acts as a tumor suppressor. DDX3 localizes to centrosomes throughout the cell cycle and prevents chromosome misalignment by inactivating and aggregating supernumerary centrosomes (Chen et al., 2017). DDX silencing subsequently led to chromosome misalignment, segregation defects, and eventually cell death. In another study, loss of DDX3 led to enhanced cell proliferation (Chang and Liu, 2010). Moreover, DDX3 localizes to the midbody during late cell division (Chen et al., 2017). These studies support the possibility of novel roles of RNA helicases in maintaining structural integrity of essential mitotic and cytokinesis components, therefore ensuring successful completion of cell division.

## Nuclear Proteins

### Importin-β

Importin-β (human transportin/karyopherin b2) acts as a transporter for proteins and complexes, moving them into the nucleus. Once thought to be exclusively a transporter, importin- β is gradually emerging as a regulator of various other cellular processes, including cell cycle and cytokinesis. For instance, a mutation in the coding gene *Kap104*, which codes for the importin-β homolog in budding yeast, promotes mitotic exit (Asakawa and Toh-e, 2002). Overexpression of importin-β also leads to aberrant spindle formation and delayed mitotic progression (Roscioli et al., 2012).

In the context of cytokinesis, importin-β might function through interacting with anillin – a direct interactor of the cytokinetic regulator RhoA and a scaffolding protein of the CN. Anillin contains a highly conserved nuclear localization signal (NLS) that binds to importin-β and is needed to mediate cortical polarization during cytokinesis; mutating its NLS significantly reduces anillin’s affinity for the equatorial cortex (Beaudet et al., 2017). Interestingly, overexpression of importin-β negatively regulates anillin’s cortical localization, instead of enhancing it, suggesting that importin-β competes with a cortical receptor for anillin binding. Thus, free importins may function as a molecular ruler, or buffer, for which an optimal level is required to maintain the appropriate cortical recruitment of anillin. Binding of importin-β is directly regulated by Ran-GTP and therefore, the potential functions of importin-β in cytokinesis are likely to be coupled with Ran and its GTP gradient, whose potential cytokinetic functions are discussed next.

### RAN

Ras-related nuclear protein (Ran) is a small G-protein involved in transporting various proteins and cellular components in and out of the nucleus through the nuclear pore complex. The function of Ran is tightly regulated by the GTP gradient across the nuclear membrane. Ran has been implicated in numerous cellular processes such as DNA synthesis, nuclear envelope structure, and cell cycle progression (Sazer and Dasso, 2000). Mounting evidence now suggests that Ran also participates in regulating cytokinesis.

Recent studies revealed that chromatin-associated signals can regulate the cortical dynamics during cytokinesis (Norden et al., 2006; Mendoza et al., 2009). Due to its concentration gradient with the peak occurring around the chromatin, chromatin-associated Ran-GTP is potentially also a regulator of the cortex. During meiosis of mouse oocytes, chromatin positioned near the cortex induces the formation of an actin cap via Ran-GTP, and the placement of DNA-coated beads near the cortex induces cortical polarity independent of microtubules (Deng et al., 2007). Active Ran regulates human anillin during anaphase and decreasing Ran-GTP leads to the ectopic localization of anillin and myosin to the cell poles and blocks proper furrowing (Beaudet et al., 2017). These studies suggested that a chromatin-associated Ran-GTP gradient may function as a molecular ruler that helps recruit and organize essential components at the cortex.

In another study, Ran-GTP was found to positively regulate the actomyosin cortex for pseudocleavage furrowing in the early *Drosophila* embryo development (Silverman-Gavrila et al., 2008). Ran controls pseudocleavage furrow organization independently of its role in regulating the microtubule cytoskeleton. Further, disruption of the Ran pathway prevented pseudocleavage furrow formation and restricted the depth and duration of furrow ingression of those pseudocleavage furrows that did form. To further elucidate the mechanism through which Ran-GTP acts to regulate cytokinesis, the authors found that Ran is required for the pseudocleavage furrow localization of the septin, peanut, a protein whose association with anillin contributes to stabilization of the CN. In fact, the direct binding of the nuclear transport receptors importin-α and -β to anillin prevented the binding of peanut to anillin (van Oostende Triplet et al., 2014), and since binding of importin-β is directly regulated by Ran-GTP, Ran appears to be a regulator of anillin and peanut’s association. Therefore, Ran-GTP plays an important role in pseudocleavage furrow ingression in syncytial embryos.

Overall, Ran-GTP acts as a microtubule-independent pathway that regulates polarization of contractile protein anillin (a process traditionally thought to require microtubules) to couple signals from the chromatin to the cortex and to ensure robustness of cytokinesis.

### Lamin B

Lamins are important constituents of the nuclear lamina in eukaryotes. Apart from their functions in maintaining the structural integrity of the nuclear pores and membranes, lamins are crucial for the assembly and disassembly of the nuclear envelope during mitosis (Lopez-Soler et al., 2001; Gruenbaum et al., 2005). In addition to its canonical roles within the nucleus, lamin B, a ubiquitously expressed type of lamin, helps regulate mitosis. In M-phase-arrested *Xenopus* egg extracts, lamin B associates with the mitotic spindles as well as the surrounding regions of the spindle. When expression of lamin B was reduced using siRNA in HeLa cells, typical spindle defects such as poor spindle morphology and lack of chromosome segregation were detected, suggesting a role for lamin B in facilitating spindle assembly (Tsai et al., 2006). In the same study, the authors also reported that lamin B is also an essential component in the formation of the mitotic matrix, allowing for tethering for other spindle assembly factors. Dominant negative forms of lamin B significantly disrupted this matrix formation, leading to severing of the mitotic spindles and cytokinesis arrest. These results indicate the significant role of lamin B in maintaining the integrity of mitotic spindles during cytokinesis. The prevailing idea to explain these observations is that the disassembled lamin B is dispersed throughout the cytoplasm during mitosis. However, a fraction of lamin B may remain associated with the mitotic spindle and/or mitotic chromosomes (Beaudouin et al., 2002). This pattern of localization of lamin B elucidates its novel role in regulating cytokinesis. Interestingly, association of lamin B with the mitotic spindle is regulated by Ran-GTP, eliciting the possibility of an entirely novel pathway of mitotic spindle regulation involving Ran and its downstream targets (Ma et al., 2007; Cavazza and Vernos, 2015). Strikingly, ESCRT-III-mediated membrane fusion facilitates reintegration of lagging chromosome fragments into micronuclei through lamin-based channels during cytokinesis in *Drosophila*, emphasizing the intricate integration between the abscission and chromosome segregation machineries during cytokinesis (Warecki et al., 2020).

## Metabolic Enzymes

### Methylmalonate-Semialdehyde Dehydrogenase

Methylmalonate-semialdehyde dehydrogenase (mmsdh) is an enzyme typically thought of being located in the mitochondria and that helps catalyze the production of propionyl- and acetyl-CoA (Kedishvili et al., 2000). Recent studies suggests a possible novel role of mmsdh in regulating cytokinesis. Previously identified in a genetic suppression screen in *Dictyostelium*, over-expression of mmsdh suppressed the dominant-negative phenotype of a myosin II phosphomimetic (Ren et al., 2014). For context, to generate force, the non-muscle myosin II must polymerize into bipolar filaments, and this assembly is regulated by heavy chain phosphorylation. The myosin II phosphomimetic mimics this phosphorylated state and impairs bipolar filament assembly (Egelhoff et al., 1993). Mmsdh promoted myosin II phosphomimetic accumulation at the cortex and cleavage furrow in *myoII* null cells, thus potentially acting as a modulator of myosin II function (Ren et al., 2014).

Importantly, mmsdh showed up again as a molecular interactor of cortexillin I and IQGAP2, core mechanosensory proteins in the contractility system (Kothari et al., 2019b). It is possible that mmsdh functions in cytokinesis through a metabolic pathway that leads to post-translational modification of several contractile enzymes, including myosin II. Mmsdh catalyzes the degradation of valine, leading to the production of propionyl-CoA. Indeed, post-transtional modifications are important in modulating proteins involved in cytokinesis. In mammalian cells, anillin and myosin IIA are acetylated during cytokinesis, and histone deacetylase (HDAC) inhibitors lead to cytokinetic defects, consistent with this concept (Chuang et al., 2010; Coulton et al., 2010; Marinova et al., 2011).

### AMP-Activated Protein Kinase

AMP-activated protein kinase (AMPK) is a highly conserved serine/threonine kinase that plays central roles in metabolic stress sensing that facilitates intracellular ATP preservation in response to energy deprivation (Hardie, 2007; Jones and Thompson, 2009). AMPK is present in the nucleus and cytoplasm, and its intracellular distribution is regulated by stress and cell growth (Kodiha et al., 2007). In addition to its role as an energy sensor, AMPK was found to act as a suppressor of cell proliferation. Enhanced activation of AMPK inhibits tumor growth and oncogenic transformation of several cancer cell lines (Zakikhani et al., 2006; Kuhajda, 2008). Many studies followed to elucidate the mechanisms of AMPK regulating cell division. Substantial evidence now suggests that AMPK plays obligatory role in chromosome segregation and cytokinesis completion. A *Drosophila* genomic screen identifies AMPK as a gene crucial for proper cell cycle progression. S2 cells lacking AMPK expression show strong defects in spindle morphology (Bettencourt-Dias et al., 2004). In human cancer-derived epithelial cells, the active form of AMPK α-catalytic subunit transiently associates with several mitotic structures including centrosomes and central spindle midzone from early stages of mitosis to cytokinetic completion (Vazquez-Martin et al., 2009). Furthermore, kinase activity of Plk1, a major regulator of mitotic progression, regulates localization and activation of AMPK at the mitotic apparatus. Pharmacological inhibition of PLK1 disrupts normal localization and phosphorylation of AMPK at its catalytic site, leading to cytokinesis failure (Vazquez-Martin et al., 2011). Studies in budding yeast reveal similar observations. Loss of Snf1, the budding yeast ortholog of AMPK, caused cells to display aberrant spindle alignment, further highlighting its role in regulating the mitotic spindle (Tripodi et al., 2018). The exact mechanisms and functions of AMPK in the context of cytokinesis regulation remain to be elucidated, but AMPK could be central to physically and temporally tethering the energy state of a cell to cell cycle progression.

## Conclusion

**FIGURE 2:**
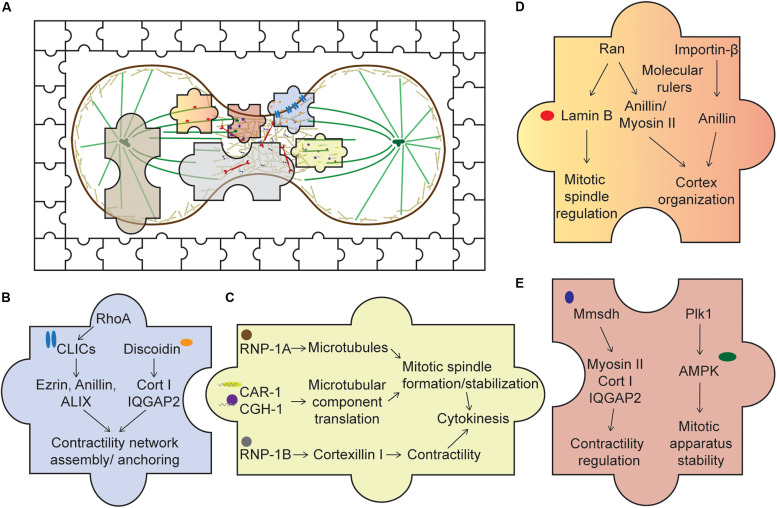
Piecing together the *unusual suspects* of the cytokinesis system. **(A)** An overview of cytokinesis with both the usual and unusual suspects, represented by the jigsaw pieces. Expanding our understanding of cytokinesis by studying these unusual suspects allows us to fully appreciate an intricate cellular system complete with cross talks, redundancy, and network integration. **(B)** Membrane-associated proteins. Upon activation of RhoA, CLICs localize to the cortex where they bind various cortical components, such as profilin-1 and ezrin. Through these interactions, CLICs may help stabilize the cortex and anchor the CN to the membrane. Discoidin, through its interaction with the actin crosslinker cortexillin I and its regulator IQGAP2, might facilitate the assembly of the contractility network. **(C)** RNA-associated proteins. RNP-1A through its protection of microtubules during cytokinesis may stabilize the mitotic spindle, ensuring successful cytokinesis. CAR-1 and CGH-1’s roles are proposed to facilitate local translation of microtubule components, ensuring proper formation of the mitotic spindle. On the other hand, RNP-1B interacts with cortexillin I and might play a role in regulating the contractility network. **(D)** Nuclear proteins. Ran and importin-β, through their interactions with various cortical proteins, such as anillin and myosin II, may act as molecular rulers, or buffers, coupled with a GTP gradient to ensure optimal recruitment of these components to the cortex. In addition, lamin B, an interactor of Ran, is crucial for mitotic spindle assembly. **(E)** Metabolic enzymes. Mmsdh appears to help myosin II function and is a biochemical interactor of cortexillin I and IQGAP2. Along with its original known biochemical activity in valine degradation, leading to the production of propionyl- and acetyl-CoA, mmsdh may promote the post-translational modification of contractile proteins, such as myosin II. AMPK is also important for mitotic apparatus assembly and regulation. Activation of AMPK by Plk1 may be important to couple cellular bioenergetic state to cell cycle progression.

While substantial progress has been made with regard to the intricacies of the CN and mitotic spindle formation and function, we are now in position to appreciate that cytokinesis results from the function of a truly integrated and collaborative cellular system (Figure 2). Further, these examples of unusual suspects in cytokinesis can help motivate us to think beyond the scope of just a few sub-cellular systems and pathways that drive a cellular process. We also should take into consideration that under physiological contexts, a cellular system is under constant environmental pressures that can be chemical and mechanical in nature. Cells are exquisitely exceptional at coping with these challenges. Recent studies have drawn upon this principle to identify new roles of known factors as well as new unknown factors. For instance, a study recently published used mechanically loaded cellular systems to study cytokinesis in the context of mechanics and identified new functions of several factors (Singh et al., 2019). The authors found that factors such as MEL-11 and LIN-5, canonically involved in regulating myosin II function and spindle positioning, respectively, are also involved in regulating cortical rotation, further elucidating how cells simultaneously respond to and create forces during cytokinesis.

Expanding our understanding of cytokinesis by studying these unusual suspects allows us to fully appreciate an intricate cellular system loaded with cross talks, redundancy, and network integration. By doing so, we will begin to better understand how other systems operate. For example, RNPs localize to and help organize P-granules, leading to the concept of phase transitions. It is also important to note that most current scientific methods still focus on cells and tissues in their most optimal culturing or physiological conditions. However, cells in their native contexts, such as in the tissue of an organism, are embedded in a complex 3D environment where they are constantly challenged with stresses and signals from their surroundings. No doubt, the concept of cellular complexes being formed through low affinity, highly dynamic, and responsive interactions is a more general underpinning of highly integrated, robust, and adaptive cellular systems and processes for which cytokinesis is a model example.

## Author Contributions

LN and DR wrote and edited the review.

## Conflict of Interest

The authors declare that the research was conducted in the absence of any commercial or financial relationships that could be construed as a potential conflict of interest.
